# 4465 Comparing Children’s Physical Activity Accumulation Between a Nature-Based and Traditional Pre-K School Setting

**DOI:** 10.1017/cts.2020.377

**Published:** 2020-07-29

**Authors:** Nathan Tokarek, Chi C. Cho, Ann M. Swartz

**Affiliations:** 1 University of Wisconsin - Milwaukee

## Abstract

OBJECTIVES/GOALS: The primary aim of this observational study was to explore minute by minute differences in children’s in-school PA accumulation while attending a nature-based compared to a traditional Pre-K program. METHODS/STUDY POPULATION: Participants from a single Pre-K program wore an accelerometer at the waist during school for two consecutive weekdays in the winter, chosen for consistent weather conditions. In this program, one day was spent at a nature-based site, and one day at a traditional classroom location. Accelerometer data was analyzed using Butte (2014) vector magnitude activity thresholds summed by minute across each day. Paired-sample t-tests were applied on a minute-by-minute basis at a significance of *p*<0.001 to determine the point(s) at which PA accumulation diverged between settings. Direct observation (DO) conducted by a trained researcher also documented activities children engaged in each school day. RESULTS/ANTICIPATED RESULTS: In-school PA differed significantly between settings beginning at minute 37 of classroom time. Based on results obtained through DO, this coincided with the end of unstructured free play time and the start of structured activities across both days. In a traditional classroom setting, structured activities included classroom-based learning, while the nature-based setting incorporated a 10-minute outdoor walk prior to the start of classroom learning. This walking period altered the trajectory of total in-school PA accumulation between program locations, with participants maintaining a significantly greater PA accumulation while in a nature-based setting through the end of the school period. DISCUSSION/SIGNIFICANCE OF IMPACT: Compared to a traditional setting, nature-based programs allow for more active structured periods in school. A 10-minute teacher-led walk can significantly improve the trajectory of children’s PA accumulation throughout the remainder of a school day.

